# Linear Interstitial Keratitis: A Report of Two Cases and Review of Literature

**DOI:** 10.7759/cureus.80985

**Published:** 2025-03-22

**Authors:** Luis C Barrientos, Michael Wildes

**Affiliations:** 1 Department of Ophthalmology, University of Colorado School of Medicine, Aurora, USA

**Keywords:** anterior segment oct, confocal microscopy, interstitial keratitis, linear interstitial keratitis, linear keratitis

## Abstract

Linear interstitial keratitis is an extremely rare variant of interstitial keratitis characterized by horizontal linear stromal infiltrates. This case series aims to expand the existing literature on linear interstitial keratitis by presenting two additional cases. The retrospective series includes two patients treated at a tertiary care hospital, both of whom underwent comprehensive clinical evaluations, including anterior segment optical coherence tomography (AS-OCT), and one patient underwent in vivo confocal microscopy (IVCM). The first patient, a 16-year-old female, presented to our center with a horizontal linear stromal opacity. She responded to topical steroids and was left with a residual scar outside of the visual axis. The second patient, a 31-year-old male with a history of Laser-assisted in situ keratomileusis (LASIK), presented with parallel linear stromal opacities. Both patients exhibited anterior stromal hyperreflectivity on AS-OCT. IVCM revealed hyperreflective needle-like structures in the first case only. Linear interstitial keratitis is a rare and poorly understood variant of interstitial keratitis. In our series, the condition responded to corticosteroid treatment. The second patient is the oldest patient in the literature to be diagnosed with linear interstitial keratitis and the only patient with a history of LASIK. Further studies with long-term follow-up and advanced diagnostic techniques are necessary to further classify the etiology of this rare condition.

## Introduction

Interstitial keratitis (IK) is a non-ulcerating, non-suppurative inflammation isolated to the corneal stroma (interstitium) without epithelial or endothelial involvement. This often presents with neovascularization and is typically the endpoint of many corneal diseases [1,2]. Linear interstitial keratitis is an extremely rare variant characterized by horizontal linear stromal infiltrates. To our knowledge, only 14 cases of linear IK have been previously described in the ophthalmic literature; however, a clear histopathologic etiology has yet to be established [3-8]. Although linear IK was previously thought to be of syphilitic origin due to positive syphilis serology in patients, subsequent cases have reported patients to be healthy with negative syphilis serology [9,10]. Most recently, Petrovic et al. described anterior segment optical coherence tomography (AS-OCT) and in vivo confocal microscopy findings (IVCM) in linear IK [6]. Additionally, Blaser et al. have described four cases of linear IK in which they performed extensive local and systemic evaluations and failed to find a cause [8]. In this series, we describe two additional cases of linear interstitial keratitis treated by one corneal specialist at a tertiary care hospital to expand our clinical knowledge of this condition.

## Case presentation

Case 1

A 16-year-old female was referred to our eye center by her local ophthalmologist for four days of pain, photophobia, redness, and foreign body sensation in the right eye. Of note, the patient mentioned this started after wearing mascara for the first time. She was evaluated by a local ophthalmologist on day zero and was instructed to start moxifloxacin 0.5% ophthalmic solution four times a day and to start prednisolone acetate 1% ophthalmic suspension four times a day, after 24 hours. Visual acuity was 20/25 in the right eye and 20/20 in the left eye on presentation. Ocular history was unremarkable except for a corneal abrasion three years prior that healed without complications, and no history of similar episodes. Family history was significant for autoimmune disease in her mother, maternal uncle, and maternal grandmother; however, specific conditions were not specified.

**Figure 1:**
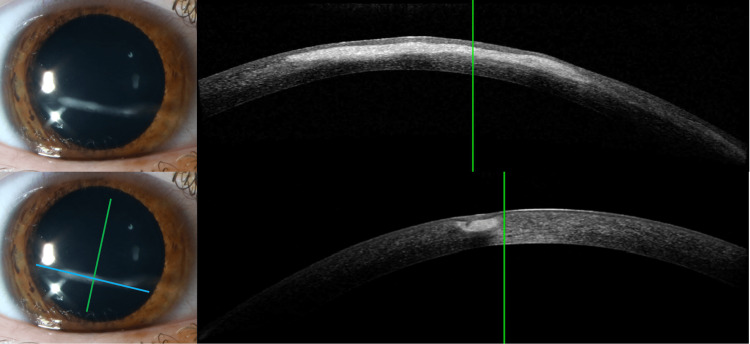
Slit Lamp and AS-OCT on Day of Presentation for Case 1 Slit lamp and anterior segment optical coherence tomography (AS-OCT) of the cornea on the day of presentation. Top left: Slit lamp image demonstrating a linear corneal opacity in 9 to 4 o’clock direction just inferior to the visual axis. Bottom left: Slit lamp image of the linear opacity with two perpendicular lines corresponding to cross-sections of AS-OCT. Top right: AS-OCT image in cross-section of opacity in the same direction (blue line) demonstrating opacity is limited to the anterior stroma. Bottom right: Perpendicular cross-section (green line) that shows opacity to be restricted to the anterior stroma.

A slit lamp examination of the right eye showed an infero-central horizontal linear stromal opacity extending from nine o’clock to four o’clock, sparing the corneoscleral limbus. There was some crystalline-like deposition, tan pigment on the nasal edge with mild neovascularization, and 30% thinning of the cornea at the nasal border. No cells were noted in the anterior chamber. Dilated fundus examination was normal. Examination of the left eye was normal. AS-OCT of the right eye revealed an opacity with a region of hyperreflectivity confined to the anterior stroma without evidence of thinning. The posterior stroma, epithelium, and endothelium appeared normal (Figure 1).

**Figure 2:**
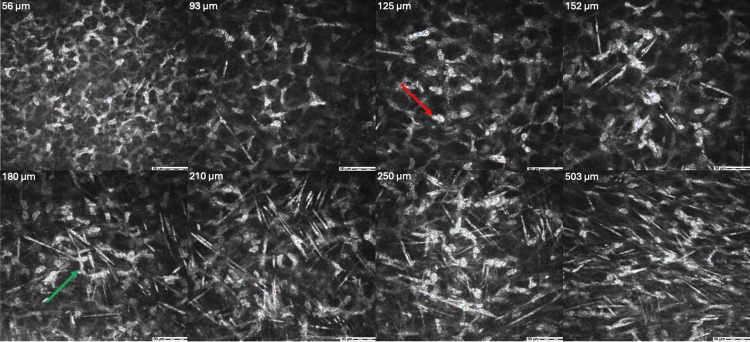
Confocal microscopy of cornea on the day of presentation for case 1 Confocal microscopy images at various depths (56-503 µm) of the stroma of the patient’s cornea. Images demonstrate hyperreflectivity of keratocytes (red arrow) and extracellular matrix mostly in the anterior stroma (56-250 µm) with some hyperreflectivity in the posterior stroma (503 µm). Hyperreflective needle-like structures (green arrow) were found throughout the stroma but were most dense in the anterior/central stroma.

Confocal microscopy revealed hyperreflectivity of keratocytes and extracellular matrix mostly in the anterior stroma but with some hyperreflectivity in the posterior stroma. Hyperreflective needle-like structures were found throughout the stroma but were most dense in the anterior/central stroma (25-271 µm) (Figure 2).

**Figure 3:**
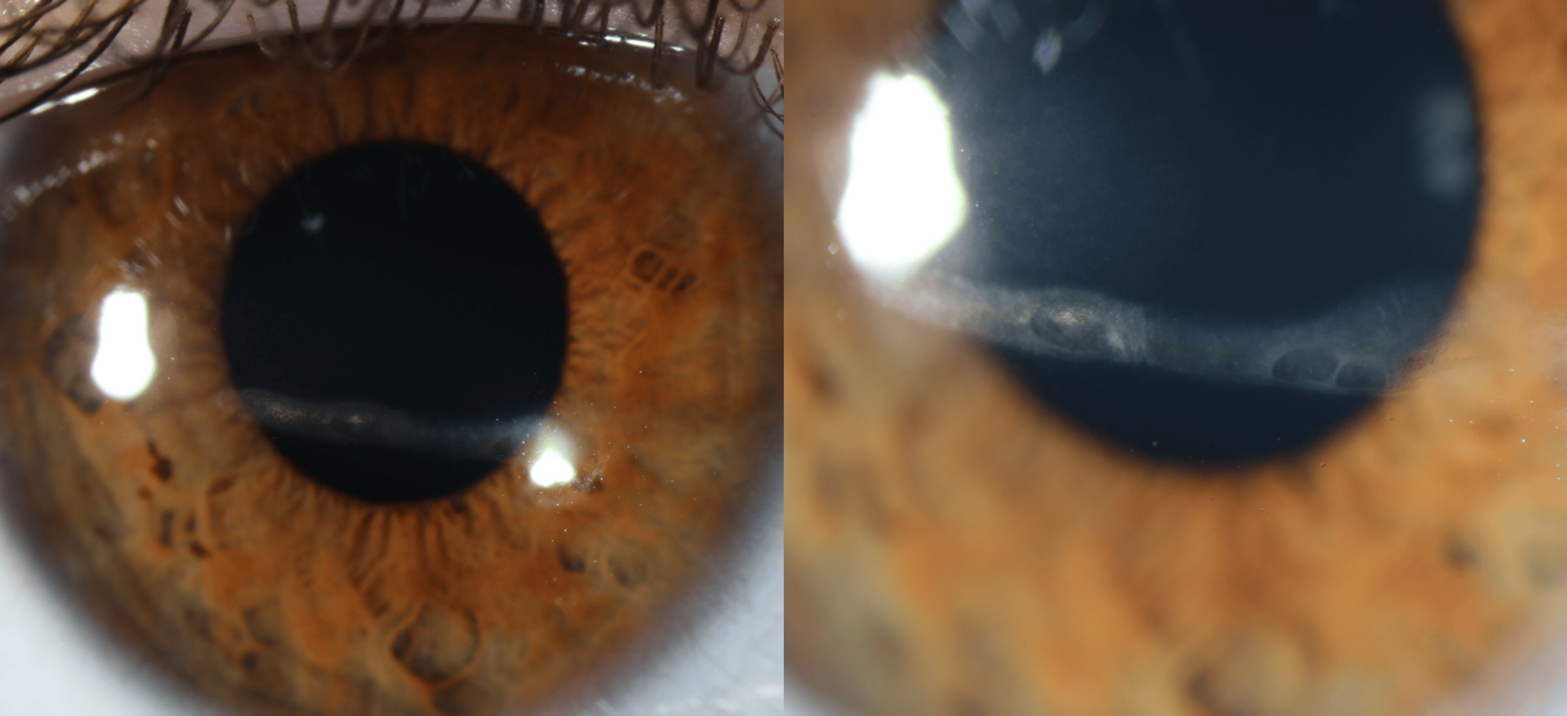
Slit lamp examination on three month follow-up for Case 1 Slit lamp images on three month follow-up. Left: Slit lamp image of linear corneal opacity in 9 to 4 o’clock direction just inferior to the visual axis. Opacification appears improved at three months. Right: Slit lamp image of the same view of the corneal opacity zoomed in to show detail of the opacity. Demonstrates improved opacification of lesions.

The patient returned for a two-week follow-up, which showed a stable exam with no further progression. At that time, she discontinued the moxifloxacin and began tapering the prednisolone acetate by one drop per week. At the seven-week follow-up, she had no further progression but was left with a residual stromal scar inferior to the visual axis (Figure 3).

Case 2

A 31-year-old male presented to our eye center for 10 days of redness, photophobia, blurred vision, and a foreign body sensation. He noted being under a significant amount of stress recently and notes that this sensation is similar to a prior episode three years ago that was treated with moxifloxacin four times daily and prednisolone acetate every two hours while awake. The visual acuity was 20/20 in the right eye and 20/20 in the left eye, but with occasional subjective blurriness. Slit lamp examination in the right eye was significant for two parallel linear anterior stromal opacities with brown pigment in the overlying epithelium. There was mild thinning of the cornea in the inferonasal area and mild adjacent neovascularization. The epithelium was intact. No cells were noted in the anterior chamber. Both eyes had LASIK flaps, blepharitis, and trace injection. The left eye was otherwise unremarkable, and the bilateral dilated fundus exam was normal. Additional ocular history included LASIK to both eyes six years prior. No significant medical or family history was noted by the patient.

**Figure 4:**
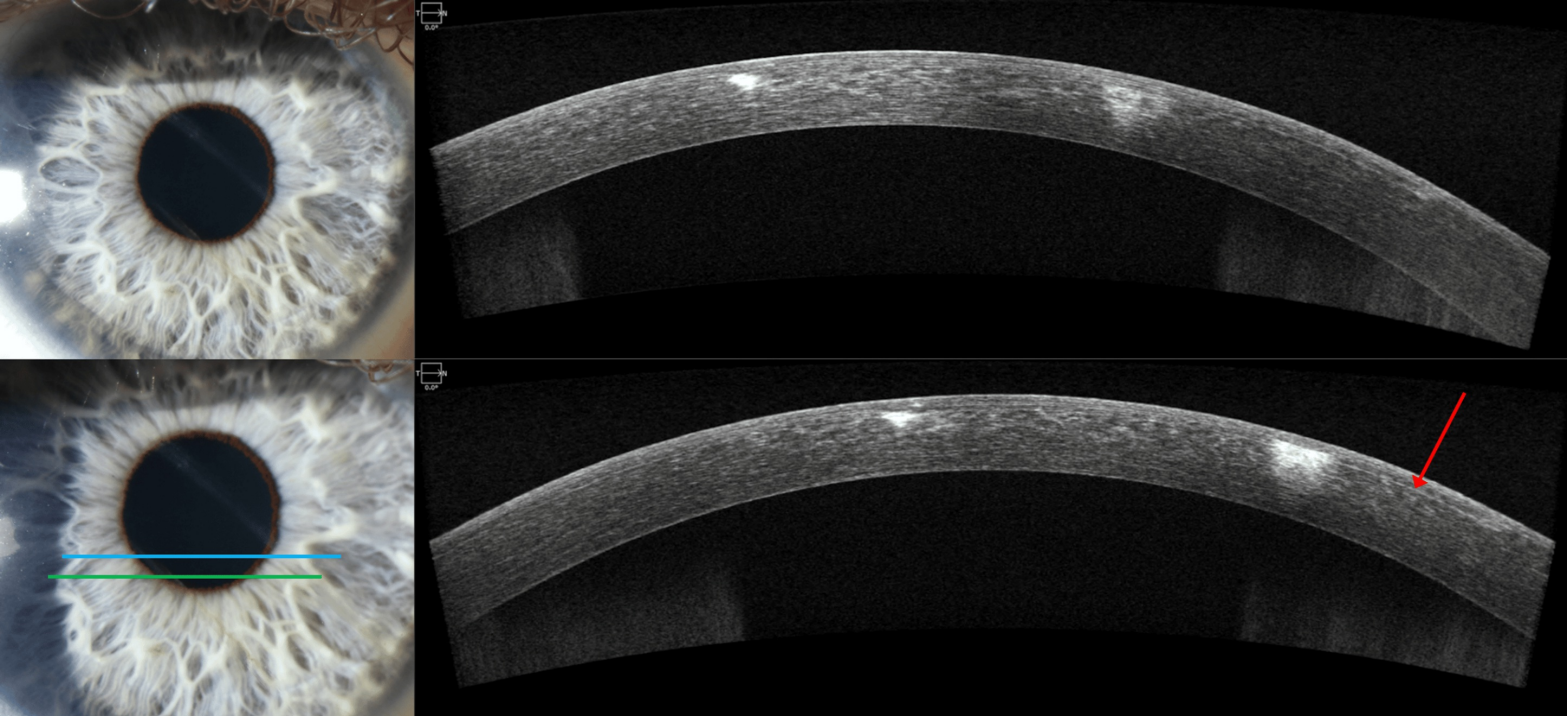
Slit lamp and AS-OCT of the right eye on the day of presentation for case 2 Slit lamp and AS-OCT of the cornea on the day of presentation. Top left: Slit lamp image demonstrating two linear corneal opacity in one extending from 5 to 10 o’clock and another from 4 to 11 o’clock. The superior opacification is in/just superior to the visual axis. Bottom left: Slit lamp image of the linear opacities with two horizontal lines corresponding to cross-sections of AS-OCT. Top right: AS-OCT image of the blue line represented in the bottom left, demonstrating two corneal opacifications limited to the anterior stroma. Bottom right: AS-OCT image of the green line represented in bottom left. The red arrow in the image represents an intact LASIK flap.

AS-OCT of the right eye revealed two regions of hyperreflectivity in the anterior stroma corresponding to the corneal opacities. The AS-OCT was aligned to be a cross-section of the two opacities seen in the cornea. The posterior stroma, endothelium, and epithelium appeared normal (Figure 4).

The patient followed up two weeks later, revealing a stable exam with no evidence of progression. At that time, he was instructed to taper the prednisolone to four times a day, then decrease it by one drop each week.

## Discussion

Linear interstitial (stromal) keratitis is a rare variant of interstitial keratitis. Since 1926, only 14 cases have been reported in the literature. Despite few cases in the literature, the clinical manifestations of linear IK were well described. In prior cases, age at onset ranged from eight to 22 years of age, with approximately half in adolescence. Most patients had resolution with topical corticosteroids, and just under half developed recurrences, with some patients having up to six episodes [6]. Lastly, some patients developed corneal scars that did not require further management unless the scar was in the visual axis [3]. In this report, one patient was 16 years old during their episode, aligning with prior reports that this condition affects mostly young patients. However, our second patient was 29 years old during the first episode and 31 years old during his second episode, making him the oldest patient in the literature to be diagnosed with linear IK. Additionally, this is the first time this clinical entity has been described in a patient with a history of LASIK.

The first cases of linear IK were described by Fuchs A [9] and Vejdovsky V [10] and initially attributed to syphilis. However, on retrospective analysis, these cases were thought not to represent linear IK as these opacities were migratory and bilateral. Additionally, subsequent cases with negative syphilis serologies were reported. In our series, neither patient had epithelial defects, and infectious etiologies were not suspected, so cultures were not obtained. Furthermore, both patients were healthy without medical issues. However, one of our patients had a history of seasonal allergies and a strong family history of autoimmune conditions spanning multiple generations. This aligns with a prior theory posed by Calvo et al. that the etiology of linear IK might be related to autoimmune conditions [3]. Although the pathogenesis remains unclear, the association between autoimmune conditions and keratitis has been well-accepted in the literature [1]. Most recently, Blaser et al. published a case series of four patients who presented with linear IK [8]. Each of their patients received a full autoimmune workup. In their series, three out of the four patients had nonspecific antinuclear antibodies (ANA) elevations; however, they were found to be diagnostically irrelevant after rheumatology consultation. In addition, they also obtained AS-OCT and IVCM, revealing anterior stromal hyperreflectivity and pan-stromal inflammation, which they postulated to be from multiple recurrences of the disease.

In our cases, AS-OCT was obtained for both patients, revealing anterior stromal hyperreflectivity similar to prior cases. IVCM was obtained in Case 1, where the same needle-like structures described by Petrovic et al. [6] were found. These structures were most dense in the anterior stroma but were seen throughout the stroma. Blaser et al. [8] suggested these findings were likely due to the multiple recurrences and natural progression of the disease. However, this was our patient’s first episode of linear IK, so it is unclear if this is a part of the underlying inflammatory process.

## Conclusions

Linear IK is a rare variant of interstitial keratitis, the etiology of which remains unknown. While it is postulated to be related to autoimmune disorders, this has yet to be confirmed. Given the recurring nature of this condition and its tendency to cause corneal scarring, we recommend rapid treatment with corticosteroids upon diagnosis of linear IK, followed by prompt follow-up to assess resolution. Additionally, we recommend educating patients on the importance of monitoring symptoms of recurrence and presenting for an evaluation as soon as they appear. Future reports could include longitudinal follow-up to evaluate for the development of autoimmune conditions, as well as additional diagnostics such as corneal sensation testing, description of the corneal nerves on IVCM, comparison of findings between both eyes and investigations for infectious processes like HSV or syphilis. By including these cases in the literature, we aim to enrich our understanding of this challenging condition.
